# Supplementary material for: Appeals to shared suffering in the context of the Israeli-Palestinian conflict
Source: PLoS One. 2025 Oct 6;20(10):e0332197. doi: 10.1371/journal.pone.0332197 (PMC12500118; doi:10.1371/journal.pone.0332197)
Supplement: S1 File — (PDF) [file pone.0332197.s001.pdf]

Appendix

Appeals to Shared Suffering in the Context of the Israeli-Palestinian Conflict

Lukas Reinhardt, Harvey Whitehouse

Corresponding authors: lukas.reinhardt@anthro.ox.ac.uk, harvey.whitehouse@anthro.ox.ac.uk

The Appendix includes:

Appendix S1: Transcript of the speech used in our study

Appendix S2: Speech that Lord John Alderdice delivered at the House of Lords on 24 October, 2023

Appendix S3: Timeline data collections

Appendix S4: Additional results study 1

Appendix S5: Additional results study 2

Appendix S6: Additional results wave 2

Appendix S7: Materials and Methods

Appendix S1: Speech that Lord John Alderdice delivered at the House of Lords on 24 October, 2023

The speech can be found on the parliamentary website of Lord John Alderdice [accessed: 13 February, 2024]: https://members.parliament.uk/member/3478/career

Appendix S2: Timeline data collections

October 7, 2023: Hamas-led attack on Israel.

December 14, 2023: Start of data collection for study 1 (open until the morning of December 17, 2023).

December 15, 2023: Start of data collection for study 2 (finished on the same day).

December 17, 2023: We invited subjects of study 1 to take our second wave (we closed data collection for wave 1 before we started wave 2). We closed data collection for wave 2 after four days.

December 18, 2023: We invited subjects of study 2 to take our second wave. We closed data collection for wave 2 after four days.

December 20, 2023: Last day of data wave 2 collection for subjects from study 1.

December 21, 2023: Last day of data wave 2 collection for subjects from study 2.

Additional information on the data collection for study 1: On the first day of data collection (December 14, 2023), we collected data from 132 participants, on the second day (December 15, 2023) we collected data from 26 participants, and on the third day (December 16, 2023) we collected data from 16 participants. We decided to stop the data collection after the third day because we did not expect many further participants to join after this day. Thus, in total we were able to collect data from 174 participants.

Appendix S3: Additional results study 1

Table S1. Correlations between outcome variables before treatment (attitudes towards Israelis)

	FusionIsraelisPre	TrustIsraelisPre	FriendshipIsraelisPre
FusionIsraelisPre	1		
TrustIsraelisPre	0.757***	1	
FriendshipIsraelisPre	0.752***	0.880***	1

* $p < 0.05$, ** $p < 0.01$, *** $p < 0.001$

Table S2. Correlations between outcome variables before treatment (attitudes towards Palestinians)

	FusionPalestiniansPre	TrustPalestiniansPre	FriendshipPalestiniansPre
FusionPalestiniansPre	1		
TrustPalestiniansPre	0.739***	1	
FriendshipPalestiniansPre	0.677***	0.879***	1

* $p < 0.05$, ** $p < 0.01$, *** $p < 0.001$

We found no significant differences between subjects who had better attitudes towards Israelis before treatment and subjects who had better attitudes towards Palestinians before treatment with regards to i) the effect of the speech on attitudes towards the ingroup (p=0.830; OLS with robust SE), and ii) the effect of the speech on attitudes towards the outgroup (p=0.589; OLS with robust SE).

Subjects reported an average psychological impact of the conflict of 3.57 which ranged between 3 (Medium impact) and 4 (Strong impact) on a scale from 1 (no impact) to 5 (extreme impact).

Turning to the three the single variables that constitute the index, we found a positive effect of the treatment on fusion with the ingroup ($M_{Fusion\ Ingroup\ Pre\ Treatment}$= 3.605; $M_{Fusion\ Ingroup\ Post\ Treatment}$= 3.697; p=0.026; two-tailed t-test) and no effects on trust towards the ingroup ($M_{Trust\ Ingroup\ Pre\ Treatment}$= 73.76; $M_{Trust\ Ingroup\ Post\ Treatment}$= 74.17; p=0.598; two-tailed t-test) and openness to friendship towards the ingroup ($M_{Friendship\ Ingroup\ Pre\ Treatment}$= 80.77; $M_{Friendship\ Ingroup\ Post\ Treatment}$= 80.46; p=0.590; two-tailed t-test).

We found positive effects on fusion with the outgroup ($M_{Fusion\ Outgroup\ Pre\ Treatment}$= 1.401; $M_{Fusion\ Outgroup\ Post\ Treatment}$= 1.743; p<0.001; two-tailed t-test), trust towards the outgroup ($M_{Trust\ Outgroup\ Pre\ Treatment}$= 28.80; $M_{Trust\ Outgroup\ Post\ Treatment}$= 31.34; p=0.004; two-tailed t-test), and openness to friendship towards the outgroup ($M_{Friendship\ Outgroup\ Pre\ Treatment}$= 33.82; $M_{Friendship\ Outgroup\ Post\ Treatment}$= 35.37; p=0.026; two-tailed t-test). These effects on the three components of the outgroup index expressed as shares of the average pre-treatment gap between the ingroup value and the outgroup value (e.g., treatment effect on fusion with outgroup

divided by the difference between pre-treatment fusion with ingroup and pre-treatment fusion with outgroup in the sample) are as follows: 15.5% for fusion, 5.7% for trust and 3.3% for openness to friendship. The difference between the fusion effect and the trust effect is significant ($p<0.001$, two-tailed t-test) while the difference between the trust effect and the openness to friendship effect is not significant ($p=0.218$, two-tailed t-test). These results are theoretically plausible since the effect of perceptions of shared transformative experiences on fusion are directly predicted by fusion theory. Also, increases in fusion and a resulting sense of caring can also quite possibly not go hand in hand with equally strong increases in trust and openness to friendship since it is possible that people care about and are fused with the outgroup in a conflict but still somewhat distrust members of the outgroup and do not want to be friends with them. An alternative reason are the different scales we used for fusion (from 1-5) and the other two outcomes (from 0-100) as the smallest movement of the fusion scale covers more distance than the smallest movement on the other two scales.

Table S3. Heterogeneous effects, OLS regression with robust standard errors. 'ChangeOGIndex' denotes changes in the outgroup index. 'Jew', 'UniEdu' and 'Female' are dummies that take the value of 1 if the participant is a Jew, university educated, or female and 0 otherwise. 'PolRight' denotes the political spectrum from 1 (Left) to 10 (Right), 'PsyImpact' the emotional impact of news about the conflict from 1 (No impact) to 5 (Extreme impact), and 'DiffPre' denotes the absolute difference between the Israeli index and the Palestinian index before exposure to the speech.

	ChangeOGIndex
Jew	-0.0230
	(0.251)
Age	0.00104*
	(0.080)
UniEdu	-0.0158
	(0.426)
Female	0.0113
	(0.424)
PolRight	-0.00353
	(0.198)
PsyImpact	-0.00356
	(0.661)
DiffPre	-0.0318
	(0.237)
Constant	0.0632*
	(0.057)
N	152
r2	0.0639

p-values in parentheses

* $p < 0.1$, ** $p < 0.05$, *** $p < 0.01$

Appendix S4: Additional results study 2

Table S4. Correlations between outcome variables before treatment (attitudes towards Israelis)

	FusionIsraelisPre	TrustIsraelisPre	FriendshipIsraelisPre
FusionIsraelisPre	1		
TrustIsraelisPre	0.419***	1	
FriendshipIsraelisPre	0.393***	0.777***	1

* $p < 0.05$, ** $p < 0.01$, *** $p < 0.001$

Table S5. Correlations between outcome variables before treatment (attitudes towards Palestinians)

	FusionPalestiniansPre	TrustPalestiniansPre	FriendshipPalestiniansPre
FusionPalestiniansPre	1		
TrustPalestiniansPre	0.412***	1	
FriendshipPalestiniansPre	0.377***	0.786***	1

* $p < 0.05$, ** $p < 0.01$, *** $p < 0.001$

We found no significant differences between subjects who had better attitudes towards Israelis and subjects who had better attitudes towards Palestinians with regards to the effect of the speech on attitudes towards the ingroup (p=0.724; OLS with robust SE). However, the effect of the speech on attitudes towards the outgroup was larger for subjects who favoured the Israeli side (diff=0.027; p= 0.017; OLS with robust SE).

Subjects reported an average psychological impact of the conflict of 2.36 that ranges between 2 (Little impact) and 3 (Medium impact) on a scale from 1 (no impact) to 5 (extreme impact). As expected, the psychological impact of the conflict on the general population was weaker than the psychological impact on Jewish and Muslim subjects (3.57).

Turning to the three the single variables that constituted the index, we found positive effects of the treatment on fusion with the ingroup ($M_{Fusion\ Ingroup\ Pre\ Treatment}$= 2.188; $M_{Fusion\ Ingroup\ Post\ Treatment}$= 2.410; p<0.001; two-tailed t-test), no effects on trust towards the ingroup ($M_{Trust\ Ingroup\ Pre\ Treatment}$= 58.24; $M_{Trust\ Ingroup\ Post\ Treatment}$= 57.89; p=0.514; two-tailed t-test) and positive effects on openness to friendship towards the ingroup ($M_{Friendship\ Ingroup\ Pre\ Treatment}$= 65.58; $M_{Friendship\ Ingroup\ Post\ Treatment}$= 63.50; p=0.001; two-tailed t-test).

We found positive effects on fusion with the outgroup ($M_{Fusion\ Outgroup\ Pre\ Treatment}$= 1.454; $M_{Fusion\ Outgroup\ Post\ Treatment}$= 1.819; p<0.001; two-tailed t-test), trust towards the outgroup ($M_{Trust\ Outgroup\ Pre\ Treatment}$= 39.52; $M_{Trust\ Outgroup\ Post\ Treatment}$= 42.92; p<0.001; two-tailed t-test), and openness to friendship towards the outgroup ($M_{Friendship\ Outgroup\ Pre\ Treatment}$=

45.27; $M_{Friendship\ Outgroup\ Post\ Treatment}$= 47.41; p<0.001; two-tailed t-test). These effects on the three components of the outgroup index expressed as shares of the average pre-treatment gap between the ingroup value and the outgroup value (e.g., treatment effect on fusion with outgroup divided by the difference between pre-treatment fusion with ingroup and pre-treatment fusion with outgroup in the sample) are as follows: 49.7% for fusion, 18.2% for trust and 10.5% for openness to friendship and the differences between these shares are significant (fusion vs trust: p<0.001, two-tailed t-test; trust vs openness towards friendship: p=0.020, two-tailed t-test). These differences are theoretically plausible since the effect of perceptions of shared transformative experiences on fusion are directly predicted by fusion theory. Also, increases in fusion and a resulting sense of caring can also quite possibly not go hand in hand with equally strong increases in trust and openness to friendship since it is possible that people care about and are fused with the outgroup in a conflict but still somewhat distrust members of the outgroup and do not want to be friends with them. An alternative reason are the different scales we used for fusion (from 1-5) and the other two outcomes (from 0-100) as the smallest movement of the fusion scale covers more distance than the smallest movement on the other two scales.

As expected, the absolute level of attitudes before treatment towards the ingroup in study 2 (0.512) was lower than in study 1 (0.732) and the absolute level of attitudes before treatment towards the outgroup in study 2 (0.320) was higher than in study 1 (0.242). The difference between the outgroup indices before and after exposure to the video of 0.049 index points had a similar size as the respective effect in the first study (0.042) suggesting that the speech was equally effective at improving attitudes towards the outgroup for Muslim and Jewish subjects and subjects from the general population.

Unsurprisingly, individual level differences were much larger for the Jewish and Muslim subjects in study 1 than for the subjects in study 2. For instance, the individual level difference between attitudes towards both sides before treatment was 0.468 index points in study 1 while it was 0.144 index points in study 2.

Table S6. Heterogeneous effects, OLS regression with robust standard errors. ‘ChangeOGIndex’ denotes changes in the outgroup index. ‘UniEdu’ and ‘Female’ are dummies that take the value of 1 if the participant is university educated, or female and 0 otherwise. ‘PolRight’ denotes the political spectrum from 1 (Left) to 10 (Right), ‘PsyImpact’ the emotional impact of news about the conflict from 1 (No impact) to 5 (Extreme impact), and ‘DiffPre’ denotes the absolute difference between the Israeli index and the Palestinian index before exposure to the speech.

	ChangeOGIndex
Age	0.000679
	(0.158)
UniEdu	-0.0226*
	(0.068)
Female	0.0199
	(0.107)
PolRight	-0.00322
	(0.259)
PsyImpact	0.0120*
	(0.052)
DiffPre	-0.0155
	(0.575)
Constant	0.0118
	(0.682)
N	271
r2	0.0578

p-values in parentheses

* $p < 0.1$, ** $p < 0.05$, *** $p < 0.01$

Appendix S5: Additional results wave 2

There were no significant differences between subjects from study 1 who dropped out and subjects from study 1 who took wave 2 with regards to attitudes before treatment towards the ingroup (p= 0.483; OLS with robust SE) and attitudes before treatment towards the outgroup (p= 0.567), although the size of the group that dropped out was certainly not large enough for a valid comparison.

In study 1, the effect on attitudes towards Israelis ($M_{Index\ Israelis\ Pre\ Treatment}$= 0.539; $M_{Index\ Israelis\ Post\ Treatment}$= 0.558; p=0.005; two-tailed t-test) was not persistent at all and went fully back to the baseline level ($M_{Index\ Israelis\ Pre\ Treatment}$= 0.539; $M_{Index\ Israelis\ Wave\ 2}$= 0.529; p=0.181; two-tailed t-test). The effect on attitudes towards Palestinians

($M_{Index\ Palestinians\ \ Pre\ Treatment}$= 0.438; $M_{Index\ Palestinians\ \ Post\ Treatment}$= 0.469; p<0.001; two-tailed t-test) was also not persistent at all and went fully back to the baseline level ($M_{Index\ Palestinians\ \ Pre\ Treatment}$= 0.438; $M_{Index\ Palestinians\ \ Wave\ 2}$= 0.447; p=0.186; two-tailed t-test). However, the positive effect on attitudes towards the outgroup and the negative effects on attitudes towards the ingroup have balanced out, leading to the pattern described above.

There were no significant differences between subjects from study 2 who dropped out and subjects from study 2 who took wave 2 with regards to attitudes before treatment towards the ingroup (p= 0.672; OLS with robust SE) and attitudes before treatment towards the outgroup (p= 0.581), although the size of the group that dropped out was also not large enough for a valid comparison.

In study 2, the effect on attitudes towards Israelis ($M_{Index\ Israelis\ Pre\ Treatment}$= 0.451; $M_{Index\ Israelis\ Post\ Treatment}$= 0.473; p<0.001; two-tailed t-test) was partially persistent although the difference between pre-treatment and wave 2 was only weakly significant ($M_{Index\ Israelis\ Pre\ Treatment}$= 0.451; $M_{Index\ Israelis\ Wave\ 2}$= 0.462; p=0.062; two-tailed t-test). The effect on attitudes towards Palestinians ($M_{Index\ Palestinians\ Pre\ Treatment}$= 0.391; $M_{Index\ Palestinians\ \ Post\ Treatment}$= 0.431; p<0.001; two-tailed t-test) was also partially persistent ($M_{Index\ Palestinians\ \ Pre\ Treatment}$= 0.391; $M_{Index\ Palestinians\ \ Wave\ 2}$= 0.410; p=0.001; two-tailed t-test).

Appendix S6: Materials and Methods

Variable Names are written in **bold script** and marked by […].

Survey that was used for Study 1 and Study 2

Please confirm:

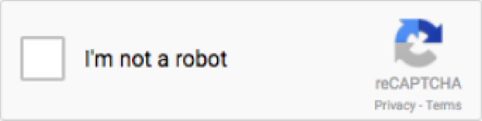

Start of Block: Consent

Informed Consent/ Assent Form

[consent] Participation in this study is voluntary and no personal data that can identify you will be collected. To participate in this study, you must be at least 18 years old.

For your participation in this study, you will receive a payment. Your participation is entirely voluntary, and you may withdraw from participation at any time during the survey, without providing any reasons. You may withdraw from the study without penalty or loss of benefits to which you may otherwise be entitled. Compensation will be awarded upon completion of the entire study. This study is conducted by a team of researchers based at the University of Oxford in the UK. Further information about your rights to information you provide is available from the University's data protection web site: https://compliance.web.ox.ac.uk/individual-rights.

In this study, we will show a video about a speech about the current armed conflict between Israel and Hamas and the situation in Gaza. Data are anonymized and only data without any personal information will be used in analysis and shared with other researchers. You will never be identified as a participant. If you have any questions, you may contact us via the Prolific chat.

This research project has been reviewed and approved by an Oxford University ethics committee. If you wish to make a formal complaint, please contact the ethics committee. Their email address is ethics@socsci.ox.ac.uk. Please indicate whether you agree to participate in this study.

- I AGREE to participate
- I DO NOT AGREE to participate

End of Block: Consent

Start of Block: Prolific ID

Welcome to this study!

In this study, we will show you a **video with sound.**

The video will include a speech by a politician about the current armed conflict between Israel and Hamas and the situation in Gaza.

If you don't want to participate in the study, you can exit the study right now.

If you want to participate, please continue to the next page.

Page Break

What is your Prolific ID?

Please note that this response should auto-fill with the correct ID

____

End of Block: Prolific ID

Start of Block: Outcome Palestinians Pre

Looking at the diagram below, please choose one of the letters (A, B, C, D, or E) shown below the picture that best represents your relationship with the group, in this case the group being **all Palestinians**.

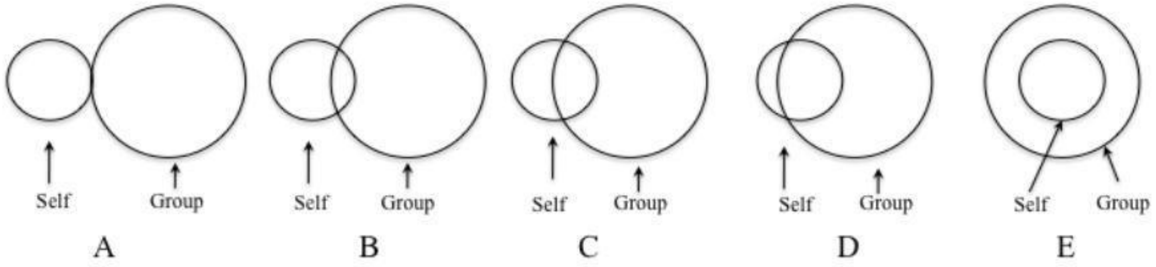

[fusionPalPre] Right now, which picture describes your relationship with **all Palestinians** best?

- ○ A
- ○ B
- ○ C
- ○ D
- ○ E

Page Break

[trustPalPre_1] How strongly would you trust **a random Palestinian**? (0 means "Do not trust at all" and 100 means "Trust completely")

	Do not trust at all	Trust completely
	0 10 20 30 40 50 60 70 80 90 100	
Your answer:		

[friendshipPalPre_1] Could you imagine becoming friends with **a random Palestinian**? (0 means "Very unlikely" and 100 means "Very likely")

	Very unlikely	Very likely
	0 10 20 30 40 50 60 70 80 90 100	
Your answer:		

End of Block: Outcome Palestinians Pre

Start of Block: Outcome Israelis Pre

Looking at the diagram below, please choose one of the letters (A, B, C, D, or E) shown below the picture that best represents your relationship with the group, in this case the group being **all Israelis**.

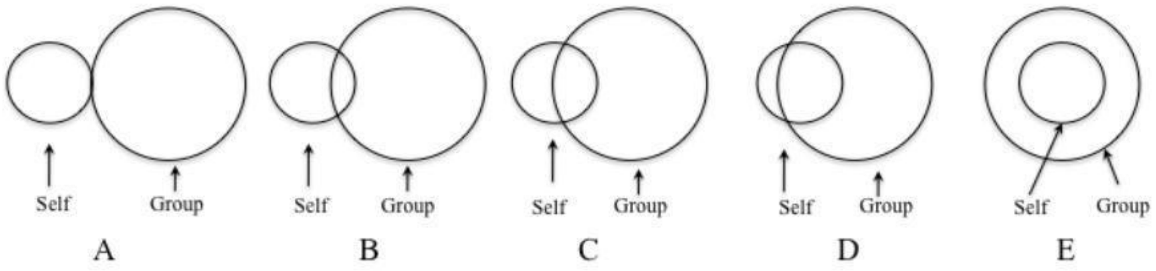

[fusionIsrPre] Right now, which picture describes your relationship with **all Israelis** best?

- ○ A
- ○ B
- ○ C
- ○ D
- ○ E

Page Break

[trustIsrPre_1] How strongly would you trust **a random Israeli**? (0 means "Do not trust at all" and 100 means "Trust completely")

	Do not trust at all	Trust completely
	0 10 20 30 40 50 60 70 80 90 100	
Your answer:		

[friendshipIsrPre_1] Could you imagine becoming friends with **a random Israeli**? (0 means "Very unlikely" and 100 means "Very likely")

	Very unlikely	Very likely
	0 10 20 30 40 50 60 70 80 90 100	
Your answer:		

End of Block: Outcome Israelis Pre

Start of Block: Fusion with Family

Looking at the diagram below, please choose one of the letters (A, B, C, D, or E) shown below the picture that best represents your relationship with the group, in this case the group being **your family**.

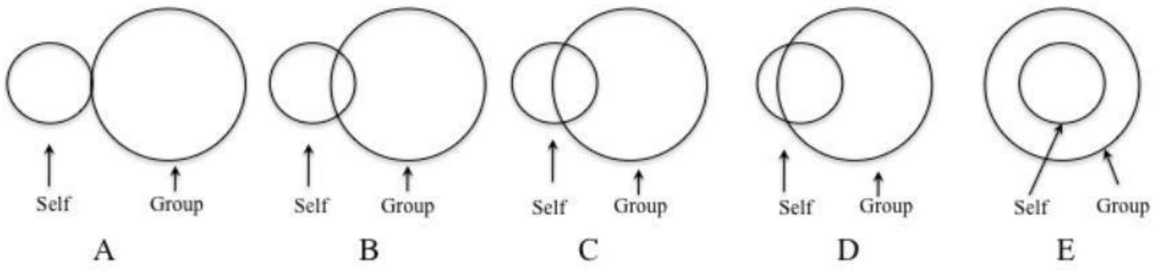

[fusionFamily] Right now, which picture describes your relationship with **family** best?

- ◯ A
- ◯ B
- ◯ C
- ◯ D
- ◯ E

End of Block: Fusion with Family

Start of Block: Psychological Impact

[psyImpact] To what extent has news reporting on violence between Israelis and Palestinians impacted you psychologically?

- ◯ Extreme impact
- ◯ Strong impact
- ◯ Medium impact
- ◯ Little impact
- ◯ No impact

End of Block: Psychological Impact

Start of Block: Demographics

[age] How old are you (in years)?

▼ 18 ... 99

[education] What is the highest level of school you have completed or the highest degree you have received?

- ○ Less than high school degree
- ○ High school degree
- ○ Bachelor's degree
- ○ Master's degree
- ○ More than master's degree

[gender] What is your gender?

- ○ Male
- ○ Female
- ○ Other

[religion] What is your religion?

- ○ Christianity
- ○ Islam
- ○ Judaism
- ○ Hinduism
- ○ Buddhism
- ○ Other religion
- ○ No Religion
- ○ My religious and spiritual beliefs are hard to classify

[politicalSpectrum_1] In political matters, people talk of "the left" and "the right." How would you place your views on this scale, generally speaking?

	Left									Right
	1	2	3	4	5	6	7	8	9	10
Your answer:										

[attentionCheck_1-4] Please indicate how strongly you agree or disagree with the following statements:

	Strongly agree	Agree	Neither agree nor disagree	Disagree	Strongly disagree
It is important to me how others think about me	○	○	○	○	○
I believe that society is based on rules and everyone must follow the rules	○	○	○	○	○
It is important to pay attention in this study. Please click "Strongly agree"	○	○	○	○	○
My family is very important to me	○	○	○	○	○

[income] What was your total household income before taxes during the past 12 months?

- ◯ Less than $25,000
- ◯ $25,000-$49,999
- ◯ $50,000-$74,999
- ◯ $75,000-$99,999
- ◯ $100,000-$149,999
- ◯ $150,000 or more
- ◯ Prefer not to say

[party] Do you lean towards a political party?

- ◯ Democrats
- ◯ Republicans
- ◯ Other party
- ◯ No party

End of Block: Demographics

Start of Block: Video

Please watch the following video from John Alderdice who is a member of the House of Lords in the United Kingdom. In the video, he gives a short speech about **the war between Israel and Hamas and the situation in Gaza.**

Please make sure to turn on the sound and watch the video from the beginning to the end.

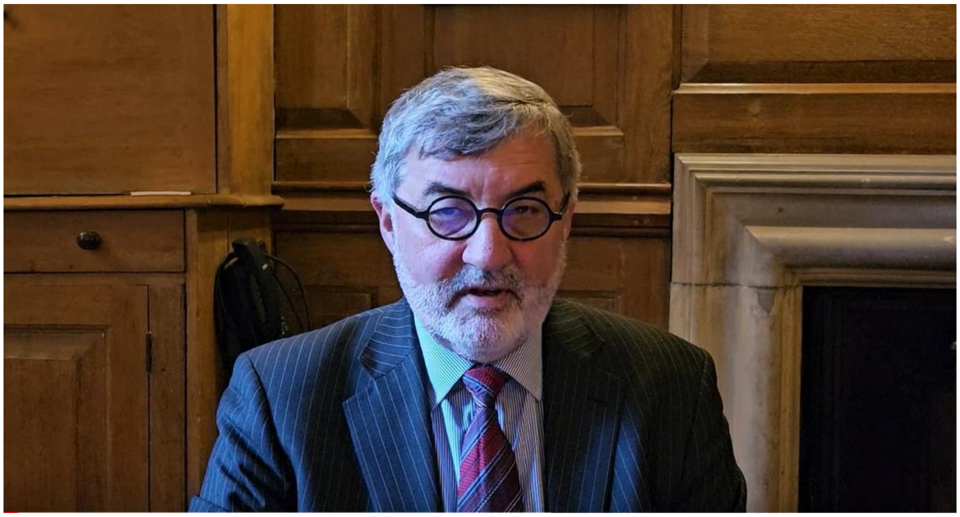

You can proceed to the next page after you have watched the whole video.

Page Break

In the following we will ask you a few questions about the video.

Please answer honestly, there are no right or wrong answers!

End of Block: Video

Start of Block: Outcome Palestinians Post

Looking at the diagram below, please choose one of the letters (A, B, C, D, or E) shown below the picture that best represents your relationship with the group, in this case the group being **all Palestinians**.

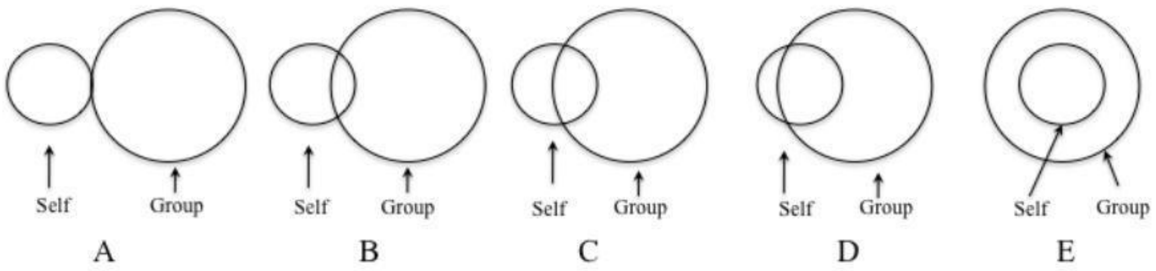

[fusionPalPost] Right now, which picture describes your relationship with **all Palestinians** best?

- ○ A
- ○ B
- ○ C
- ○ D
- ○ E

Page Break

[trustPalPost_1] How strongly would you trust **a random Palestinian**? (0 means "Do not trust at all" and 100 means "Trust completely")

	Do not trust at all	Trust completely
	0 10 20 30 40 50 60 70 80 90 100	
Your answer:	[slider]	

[friendshipPalPost_1] Could you imagine becoming friends with **a random Palestinian**? (0 means "Very unlikely" and 100 means "Very likely")

	Very unlikely	Very likely
	0 10 20 30 40 50 60 70 80 90 100	
Your answer:		

End of Block: Outcome Palestinians Post

Start of Block: Outcome Israelis Post

Looking at the diagram below, please choose one of the letters (A, B, C, D, or E) shown below the picture that best represents your relationship with the group, in this case the group being **all Israelis**.

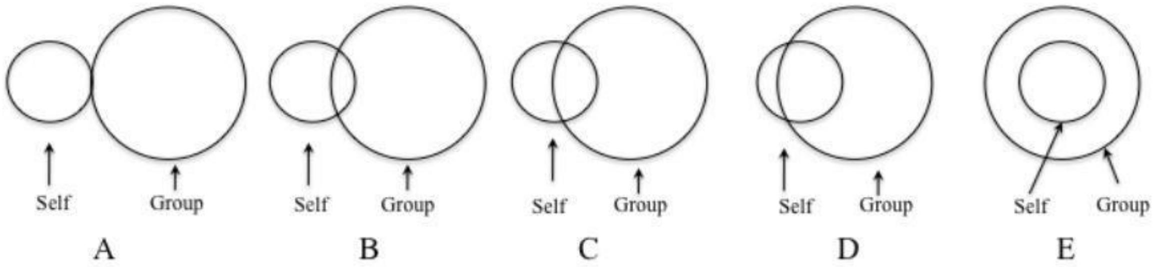

[fusionIsrPost] Right now, which picture describes your relationship with **all Israelis** best?

- ○ A
- ○ B
- ○ C
- ○ D
- ○ E

Page Break

[trustIsrPost_1] How strongly would you trust **a random Israeli**? (0 means "Do not trust at all" and 100 means "Trust completely")

	Do not trust at all									Trust completely	
	0	10	20	30	40	50	60	70	80	90	100
Your answer:											

[friendshipIsrPost_1] Could you imagine becoming friends with **a random Israeli**? (0 means "Very unlikely" and 100 means "Very likely")

	Very unlikely									Very likely	
	0	10	20	30	40	50	60	70	80	90	100
Your answer:											

End of Block: Outcome Israelis Post

Start of Block: Comprehension Check

[comprehensionCheck] What was the main argument of the speech?

- ○ The UK government should support Israel
- ○ The UK government should support Palestine
- ○ Civilians on both sides suffer which should motivate peace efforts
- ○ Only higher investments in the military can guarantee peace

End of Block: Comprehension Check

Start of Block: Technical check

[technicalWatch] Did you watch the whole video?

- ○ Yes
- ○ No

[technicalSound] Did you encounter any problems regarding the sound?

- ○ Yes
- ○ No

[technicalGeneral] Did you encounter any technical problems?

- ○ Yes
- ○ No

End of Block: Technical check

Start of Block: Bug report

[bugReport] Please describe any technical problems you encountered:

____

End of Block: Bug report

Survey that was used for Wave 2

Variable Names are written in **bold script** and marked by […].

Start of Block: Consent

Please confirm:

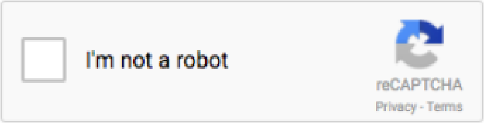

Page Break

Informed Consent/ Assent Form

[consent] Participation in this study is voluntary and no personal data that can identify you will be collected. To participate in this study, you must be at least 18 years old.

For your participation in this study, you will receive a payment. Your participation is entirely voluntary, and you may withdraw from participation at any time during the survey, without providing any reasons. You may withdraw from the study without penalty or loss of benefits to which you may otherwise be entitled. Compensation will be awarded upon completion of the entire study. This study is conducted by a team of researchers based at the University of Oxford in the UK. Further information about your rights to information you provide is available from the University's data protection web site: https://compliance.web.ox.ac.uk/individual-rights.

In this study, we will ask some questions related to the current armed conflict between Israel and Hamas and the situation in Gaza. Data are anonymized and only data without any personal information will be used in analysis and shared with other researchers. You will never be identified as a participant. If you have any questions, you may contact us via the Prolific chat.

This research project has been reviewed and approved by an Oxford University ethics committee. If you wish to make a formal complaint, please contact the ethics committee. Their email address is ethics@socsci.ox.ac.uk. Please indicate whether you agree to participate in this study.

- ◯ I AGREE to participate
- ◯ I DO NOT AGREE to participate

End of Block: Consent

Start of Block: Prolific ID

Welcome to this study!

What is your Prolific ID?

Please note that this response should auto-fill with the correct ID

____

End of Block: Prolific ID

Start of Block: Outcome Palestinians Long

Looking at the diagram below, please choose one of the letters (A, B, C, D, or E) shown below the picture that best represents your relationship with the group, in this case the group being **all Palestinians**.

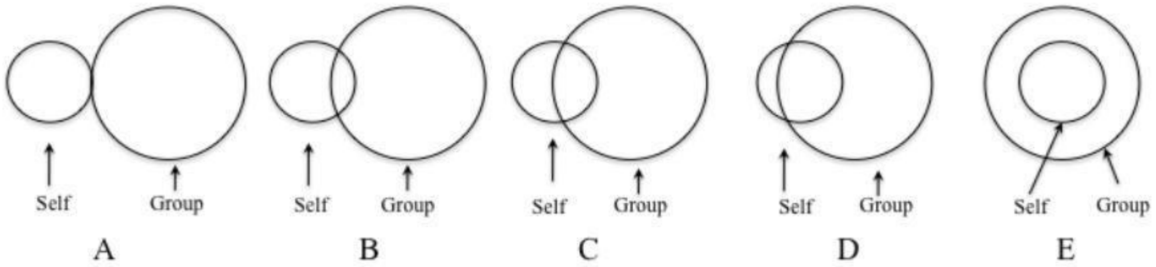

[fusionPalLong] Right now, which picture describes your relationship with **all Palestinians** best?

○ A

○ B

○ C

○ D

○ E

Page Break

[trustPalLong_1] How strongly would you trust **a random Palestinian**? (0 means "Do not trust at all" and 100 means "Trust completely")

	Do not trust at all	Trust completely
	0 10 20 30 40 50 60 70 80 90 100	
Your answer:		

[friendshipPalLong_1] Could you imagine becoming friends with **a random Palestinian**? (0 means "Very unlikely" and 100 means "Very likely")

	Very unlikely	Very likely
	0 10 20 30 40 50 60 70 80 90 100	
Your answer:		

End of Block: Outcome Palestinians Long

Start of Block: Outcome Israelis Long

Looking at the diagram below, please choose one of the letters (A, B, C, D, or E) shown below the picture that best represents your relationship with the group, in this case the group being **all Israelis**.

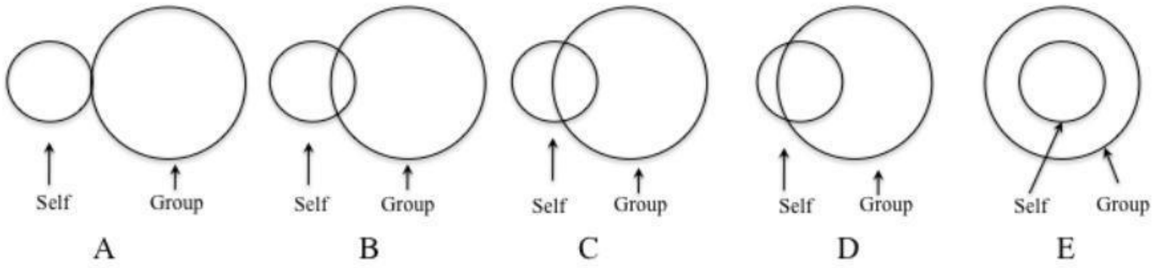

[fusionIsrLong] Right now, which picture describes your relationship with **all Israelis** best?

- ◯ A
- ◯ B
- ◯ C
- ◯ D
- ◯ E

Page Break

[trustIsrLong_1] How strongly would you trust **a random Israeli**? (0 means "Do not trust at all" and 100 means "Trust completely")

	Do not trust at all	Trust completely
	0 10 20 30 40 50 60 70 80 90 100	
Your answer:		

[friendshipIsrLong_1] Could you imagine becoming friends with **a random Israeli**? (0 means "Very unlikely" and 100 means "Very likely")

	Very unlikely									Very likely	
	0	10	20	30	40	50	60	70	80	90	100
Your answer:											

End of Block: Outcome Israelis Long
