# Supplementary material for: Appeals to shared suffering in the context of the Israeli-Palestinian conflict
Source: PLoS One. 2025 Oct 6;20(10):e0332197. doi: 10.1371/journal.pone.0332197 (PMC12500118; doi:10.1371/journal.pone.0332197)
Supplement: Attachment — Submitted filename: PLOS One Lewin 020325.docx [file pone.0332197.s010.docx]

**Appeals to Shared Suffering in the Context of the Israeli-Palestinian Conflict**

The paper presents a noteworthy and innovative research concept, with an original experimental design. The effort to identify real-world mechanisms for reducing intergroup hostility is particularly commendable. Overall, the article appears well-suited for publication.

However, I have some reservations regarding the selection of Lord John Alderdice as the speaker. Firstly, the paper does not provide a detailed account of the selection process, including the criteria used, alternative candidates considered, and the specific rationale for choosing Alderdice as the optimal speaker. Secondly, the significance of introducing an Irish figure to an American audience warrants further discussion. Would it not be more effective to present a speaker whose background and perspectives might resonate more closely with the local audience? Thirdly, given Alderdice’s selection, the paper would benefit from a more comprehensive discussion of his biography and professional standing. Beyond serving as a justification for his inclusion, such information contributes to the transparency and accountability of the research. Finally, while the inclusion of a video link to the speech is valuable, it would be advisable to also provide a written transcript of the speech for accessibility and reference.

Eyal Lewin – Ariel University
