# Supplementary material for: Appeals to shared suffering in the context of the Israeli-Palestinian conflict
Source: PLoS One. 2025 Oct 6;20(10):e0332197. doi: 10.1371/journal.pone.0332197 (PMC12500118; doi:10.1371/journal.pone.0332197)
Supplement: Attachment — Submitted filename: Response to Reviewers.docx [file pone.0332197.s011.docx]

**Response to reviewers**

We thank both reviewers for their time and their constructive and valuable feedback. We respond to their comments below.

**Comments from Reviewer #1**

Major:

**Comment 1:** Abstract: please add a description of the research sample and main results (e.g., p – values).

**Response:** We have now added information about the sample sizes of both studies in the abstract to complement our descriptions of the samples (i.e. ‘Muslim and Jewish Americans’ in the first study and ‘sample of the general US population’ in the second study). We have also noted that exposure to the speech fostered identity fusion, trust and openness to friendship towards the outgroup rather than just referring to attitudes. Since our results are highly significant and it’s somewhat unusual in our fields to state p-values in the abstract we would prefer to refrain from doing so unless this is a strict requirement.

**Comment 2:** Page 4, line 10: Authors declare: “we chose a within-subjects design” can you elaborate more about this restriction? How many participants are needed for between-subject design? As such an analysis can provide information about the different impact of the intervention (i.e., the speech) across the two ethnic groups.

**Response:** We now explain in the section ‘Introduction/Present research’ that a within-subjects design increases power because it creates two rather than one data point per participant and controls for individual-specific effects by design (Charness et al., 2012). In order to obtain the same power, between-subjects designs can require four times as many participants as within-subjects designs, although this ratio can be higher or lower depending on the relevant parameters (List, 2025). We also observe that the within-subjects design allows us to obtain treatment effects on an individual level which facilitates the analysis of heterogeneous treatment effects even in relatively small samples.

List, J. A. (2025). The Experimentalist Looks Within: Toward an Understanding of Within-Subject Experimental Designs (No. w33456). *National Bureau of Economic Research Working Paper Series*.

Charness, G., Gneezy, U., & Kuhn, M. A. (2012). Experimental methods: Between-subject and within-subject design. *Journal of economic behavior & organization*, *81*(1), 1-8.

We also added exploratory analyses of heterogeneous effects in the sections ‘Study 1/Results’ and ‘Study 2’. In both studies, we found no evidence of heterogeneous effects with regard to age, education, gender, position on the political spectrum, psychological impact of news reporting about the conflict, and pre-treatment index differences. We found significant effects of similar size for Muslims and Jews in study 1 and significant effects even for participants who were highly polarized before exposure to the speech in both studies. These results suggest that the speech is effective across a broad range of audiences.

**Comment 3:** Page 4, 12-15 - I think it will be valuable to provide a more detailed analysis of the scale that was consolidated from three distinct measures or facets (namely, identity fusion, trust and openness to friendship). The measure was called by authors as “attitudes” but actually refer to different things. Did the authors conduct a statistical analysis for each facet? It would be interesting to know which one was mostly affected by the intervention.

**Response:** We agree that the effects on three single outcome variables are interesting. We present correlations between these three variables in tables S1, S2, S4 and S5 in the Appendix and observe highly significant and strong correlations. We also present effects on the single outcome variables in study 1 and study 2 in Appendix S3 and Appendix S4 and we found significant effects for all three variables. We have now added remarks about these results in the main manuscript at the end of section ‘Study 1/Results’ and section ‘Study 2’.

**Comment 4:** Page 6, line 23-25: I am unclear about the relevance of mentioning the number of participants for each of the three days. Was there distinct or significant news about the war during these days? Did you examine the variations across them? It appears to me that you did not. Therefore, I recommend only mentioning the total sample after the three days.

**Response:** We indicated the number of participants who took part on each date to illustrate that we stopped data collection after day 3 because we did not expect many further participants to join after this day. We explain this before we turn to describing the sample characteristics in section ‘Study 1/Data’.

**Comment 5:** Results: Page 6. I believe providing a demographic description and an analysis of the differences between the groups (American Muslim vs. American Jewish participants), particularly concerning age and gender, would be valuable. If significant differences between the groups are identified, you should consider controlling for them in subsequent analyses.

**Response:** We have now added further information about differences between Jewish and Muslim participants at the end of section ‘Study 1/Data’. Jewish participants were significantly older, more left-wing, and reported less psychological impact of news reporting on the violence between Israelis and Palestinians. There were no significant differences regarding the shares of university educated participants or female participants. However, we also now emphasize that comparisons between Jewish and Muslim participants are not our focus. Moreover, in the one comparative analysis we added we found very similar effects of exposure to the speech on the outgroup index for both groups (Effect of speech on outgroup index among Jewish participants: 0.042; Effect of speech on outgroup index among Muslim participants: 0.043; p-value of the t-test comparing effect sizes between both groups: p=0.955). See also our response to your second comment where we explain that we did not find evidence for heterogeneous treatment effects with regard to demographic characteristics.

**Comment 6:** Page 7: The choice between a one-tailed or two-tailed test hinges on whether the research hypothesis predicts a specific direction for the effect, which necessitates a one-tailed test, or merely indicates that there is a difference, requiring a two-tailed test. is there a particular reason for selecting the two-tailed t-test instead of the one-tailed t-test?

**Response:** This point is well taken, and we now report one-tailed t-tests for analyses that test our directional hypotheses.

**Comment 7:** Additionally, the t-test results do not completely report the t-value.

**Response:** We now also report t-values.

**Comment 8:** The authors clearly understand the intense emotional conflict involved. I wonder if they have considered measuring the participants' emotional involvement, such as experiencing emotional distress or having family or friends injured in the conflict. These variables could add unique insights to the characteristics of this population.

**Response:** We included a psychological impact measure in the demographics block of the survey before participants were exposed to the speech. The question was ‘To what extent has news reporting on violence between Israelis and Palestinians impacted you psychologically?’ with answers ‘No impact’ (1), ‘Little impact’ (2), ‘Medium impact’ (3), ‘Strong impact’ (4), and ‘Extreme impact’ (5). Participants in study 1 (Jews and Muslims) reported an average impact of 3.57 and participants from the general US population (study 2) reported an average impact of 2.36. We have added now remarks on this in the second paragraph of section ‘Study 1/Data’ and in the third paragraph of section ‘Study 2’.

**Comment 9:** Discussion: Although the introduction is well-structured, the discussion requires further elaboration. How does this work contribute to existing literature and theoretical understanding? Additionally, consider how other communication tools might help in resolving such conflicts, perhaps by facilitating dialogue, promoting understanding, or easing tensions between parties.

**Response:** We elaborate further on our contribution to the literature in the Discussion. First, we discuss our contribution to the identity fusion literature. According to identity fusion theory, threats to the ingroup are taken personally by fused participants motivating extreme forms of pro-group action to protect the ingroup from threat which can motivate outgroup hostility. We contribute to the fusion literature by showing that highlighting suffering of ingroup and outgroup simultaneously does not necessarily lead to exacerbated intergroup conflict but can also help to heal divisions. We also elaborate that we contribute to the literature on transforming competitive victim beliefs into inclusive victim beliefs in the context of the Israeli-Palestinian conflict by demonstrating that speeches can effectively facilitate such a transformation. Finally, we situate our work within a literature on interventions that reduce animosity towards a political outgroup and discuss other tools that may also be designed for this purpose including (among others) correcting misperceptions about the outgroup, highlighting common interests, and highlighting commonalities.

**Comment 10:** It would be valuable to elaborate on the implications of the research on the real world conflict. What the authors think the effect of exposure to such speech on Israelis (directly injured vs. not directly injured in both side, e.g., Israelis that live in the center of Israel vs Gaza Envelope / Palestinians living in their permanent house vs those that were evacuated due to the IDF invasion to Gaza). How other conflicts can benefit from the research (e.g., the Ukraine – Russia war, animosity between ethnic groups in Africa esc.)

**Response:** We have now added some remarks to address this point in the fourth paragraph the Discussion after our discussion of the limitation that we only include US participants. The cross-cultural ubiquity of the link between perceptions of shared dysphoric experiences and intense group bonding as well as the fact that we find effects even for strongly polarized participants in our samples can be seen as arguments that similar interventions might also work in Israeli and Palestinian samples and in the context of other conflicts. However, we also note that future research might test the impact of similar interventions in these samples. We also note that future research might look at heterogeneous effects with regard to the degree of direct personal loss suffered in the conflict.

Minor:

**Comment 11:** Page 2, Line 39: consider deleting the words “the power of” in order to keep the tone descriptive.

**Response:** We have adopted this suggestion.

**Comment 12:** The additional files attached to the manuscript, such as .do or .dta files, are not accessible. Is there an alternative way to approach them? Additionally, the PDF supplementary file is well structured, consisting of seven sections. I recommend labeling each section as Appendix 1, Appendix 2, etc. Furthermore, while the manuscript refers readers to Appendices 1, 2, and 6, please consider directing them to the other important information available in Appendices 3, 4, 5, and 7.

**Response:** We will re-upload these files as part of our resubmission in case there were any technical issues with the files previously uploaded. Reviewer 2 indicated that the data was available to him. That said, we used Stata for the analyses and the .do file can also be opened with a text-editor and the .dta files can be imported and converted into other formats with other data analysis programmes. As recommended, we have also re-labelled the Appendix sections, and we are now referring to all Appendices in the main text. As recommended by reviewer 2, we have moved the transcript into the main manuscript, thus we have now 6 sections in the Appendix rather than 7.

**Comments from Reviewer #2**

**Comment 1:** The paper presents a noteworthy and innovative research concept, with an original experimental design. The effort to identify real-world mechanisms for reducing intergroup hostility is particularly commendable. Overall, the article appears well-suited for publication.

However, I have some reservations regarding the selection of Lord John Alderdice as the speaker. Firstly, the paper does not provide a detailed account of the selection process, including the criteria used, alternative candidates considered, and the specific rationale for choosing Alderdice as the optimal speaker. Secondly, the significance of introducing an Irish figure to an American audience warrants further discussion. Would it not be more effective to present a speaker whose background and perspectives might resonate more closely with the local audience? Thirdly, given Alderdice’s selection, the paper would benefit from a more comprehensive discussion of his biography and professional standing. Beyond serving as a justification for his inclusion, such information contributes to the transparency and accountability of the research. Finally, while the inclusion of a video link to the speech is valuable, it would be advisable to also provide a written transcript of the speech for accessibility and reference.

**Response:** We added a paragraph about Lord Alderdice’s background in section ‘Study 1/Design and hypotheses’. Lord Alderdice is a member of the House of Lords and the Select Committee of the House on International Relations and Defence. He also played a significant role in the Irish Peace Process as a former leader of the Alliance Party of Northern Ireland. We selected him as a speaker because of his substantial expertise on intractable conflicts. We acknowledge that he might be perceived differently than a US speaker by a US audience. However, we also note that this feature of our design allows us to rule out interaction effects with partisan affiliation or prior beliefs about the speaker. Thus, our setup might be in a position to investigate the impact of the speech per se without these additional layers. We have also added a remark in the fourth paragraph of the Discussion that future research might investigate whether characteristics of the speaker affect the impact of the speech, and we agree that the characteristics of the speaker are an interesting aspect of the study design. We have included a written transcript of the speech in section ‘Study 1/Transcript of the speech’ of the main manuscript.
