# Supplementary material for: Appeals to shared suffering in the context of the Israeli-Palestinian conflict
Source: PLoS One. 2025 Oct 6;20(10):e0332197. doi: 10.1371/journal.pone.0332197 (PMC12500118; doi:10.1371/journal.pone.0332197)
Supplement: Attachment — Submitted filename: Response_to_Reviewers_auresp_2.docx [file pone.0332197.s012.docx]

**Response to reviewers (second round)**

We thank both reviewers for their time and their constructive and valuable feedback. We respond to their comments below.

**Comments from Reviewer #1**

**Comment 1:** On page 5, the authors state: “The individual pictured in Fig 1 has provided written informed consent (as outlined in PLOS consent form) to publish their image alongside the manuscript.” I recommend revising this for clarity and consistency: “The individual pictured in Figure 1 has provided written informed consent (as outlined in the PLOSOne consent form) to publish his image alongside the manuscript.”

**Response:** We were advised by Plos One staff to include this sentence, so we feel we should stick to the wording provided to us.

**Comment 2:** As noted in the first round of review, please consider including the main results in the abstract. You have added the sample size, which is helpful, but including the key outcomes and p-values would be more beneficial for readers who are screening the abstract of your manuscript.

**Response:** We do already mention that the key outcomes were fusion, trust and openness to friendship. However, we don’t have enough space in the abstract to explain the index we built using these three single outcome variables in an appropriate manner, so including p-values for index results would potentially cause confusion. Also, we prefer not to report p-values for the single index components as this is not what we pre-registered.

**Comments from Reviewer #3**

**Introduction – Background on the War**: The opening paragraph, which provides a chronological overview of recent events in the Israel-Gaza war, could be significantly shortened. I am uncertain about the relevance of this historical background for the reader, who is likely already familiar with the context. Additionally, although the authors make a commendable effort to present the events in a neutral and objective tone, some readers who strongly identify with either the Israeli or Palestinian side may perceive the description as biased or unfair. Given the ongoing nature of the war and the emotional sensitivity of the topic, this paragraph risks alienating readers and undermining trust in the research. I recommend either substantially condensing the paragraph or removing it altogether, and beginning the introduction with the theoretical background—which, in my view, should be the core of the introduction.

**Response:** We agree that discussing the background of the conflict is an extremely challenging task and also that the introductory text could be shortened. In the revised version, we have deleted the second paragraph discussing divisions within the US over the Israeli-Palestinian conflict. However, we would like to retain the first paragraph because we think that giving a short description on the reactions of the relevant international institutions to the conflict is the most even-handed approach. Many readers who are emotionally impacted by the conflict could perceive as biased and unfair a version of the article that does not even mention well-documented allegations of war crimes and crimes against humanity levelled at both sides. We are careful to refer only to official processes within internationally recognized institutions without making any value judgements on these processes. We have also deleted some remaining adjectives in the first paragraph that could possibly be interpreted as value laden.

**Reporting Differences Across Outcome Dimensions:** While I understand the rationale for creating a composite outcome index from identity fusion, trust, and openness to friendship (and I acknowledge the high intercorrelations reported in the appendix), the three variables represent conceptually distinct constructs. It would be valuable to examine whether the speech had different effects across these dimensions. If the effects were similar, the authors could briefly note this in the text and include the detailed results in the appendix. Either way, some comment in the main text would help clarify the distinctiveness (or lack thereof) of the outcome components.

**Response:** We note at the very end of sections “Study 1 – Results” and “Study 2” that we discuss the effects on the single components in the Appendix (Appendix S3 for study 1 and Appendix S4 for study 2). In both studies, the effects on all three components of the outgroup index were significant. At the end of Appendices S3 and S4 we now also state the effects on all three components of the outgroup index as shares of the pre-treatment gap of the ingroup value of the component and the outgroup value of the component to make the three effects comparable. In both studies we found that the effect on fusion was significantly larger than the effect on trust and the effect on openness to friendship. These results are theoretically plausible since fusion theory predicts primarily the treatment effect on fusion. Moreover, fusion with and a resulting sense of caring for the outgroup can plausibly exist in the context of a complex conflict without equally strong levels of trust or even openness to friendship. However, we also used different scales to measure fusion (from 1-5) and the other two outcomes (from 0-100) and the smallest movement on these scales is larger for the fusion scale which could also explain the observed patterns. Therefore, we would not give these results too much weight.

**Effect Size Reporting:** The results section would benefit from more explicit reporting of effect sizes, such as Cohen’s d. While the authors describe mean changes and provide p-values, including standardized effect sizes would enhance the interpretability and transparency of the findings, especially given the within-subject design.

**Response:** We now also report Cohen’s dz.

**Daily Sample Size Reporting:** Echoing Reviewer 1’s comment, I too found the reporting of the number of participants recruited on each specific day to be puzzling. While the authors offer an explanation, it still seems unnecessary and may distract from the main narrative. I suggest removing this detail or, if retained, relocating it to a footnote or appendix.

**Response:** We moved this detail to Appendix S2 and no longer discuss it in the main text.

**Justification for Within-Subject Design:** The manuscript currently includes justification for the within-subject design in two separate locations. While the explanation is sound, it may be unnecessarily lengthy and slightly repetitive. I understand that this likely responds to Reviewer 1’s request for elaboration, but a more concise explanation in a single location would suffice. Moreover, the authors emphasize the limitations of the design, but could also briefly mention its advantages—for example, enhanced statistical power and control for individual differences.

**Response:** We have deleted the second superfluous justification of the within-subject design in section “Study 1 - Design and hypotheses”. We discuss advantages of within-subject designs in the “Present research” section.

**Power Analysis:** I suggest that the authors briefly address the absence of a priori power analysis. Although the within-subject design improves power, it would be helpful to explain why no power calculation was conducted during the planning stage.

**Response:** We have added the following text in the first paragraph of section “Study 1 – Data”: “We did not conduct an a-priori power analysis because it seemed unlikely that we would be able to recruit the full n=400 sample due to the limited number of available Jewish and Muslim participants registered on Prolific. Also, we considered n=400 to be a sufficient upper bound given our prior experiences with within-subject designs.”

**Durability of the Effect:** One additional point relates to the observation that the effects of the intervention persisted three days after exposure. While this is mentioned in both the abstract and results, the discussion section does not elaborate on the theoretical or practical implications of this finding. What does it mean, theoretically or in applied terms, that such effects last for three days? Moreover, I would caution against characterizing this as a "lasting" or "persistent" effect without qualification. Measuring the outcomes again only three days after the intervention offers a rather limited window into durability. It would be valuable for the authors to acknowledge this limitation more explicitly and, if possible, to suggest future research directions that examine longer-term impact. Even a brief discussion of these considerations would help contextualize the findings more appropriately.

**Response:** We have added a paragraph in the “Discussion” section on this (the sixth paragraph starting with “A third limitation…”). We argue that partially persistent effects of a single exposure to a 3-minute speech in video format provides evidence for the effectiveness of the approach given that participants were likely exposed to many real-world stimuli between waves in the heated and emotionally intense phase of the conflict in December 2023. We also argue that this result is consistent with identity fusion theory, and we underline the need for future research that analyses effects over longer time periods. We also recommend further research on the long-term effects of repeated exposure to interventions or exposure to even more intense interventions.
